# A test of a triadic conceptualization of future self-identification

**DOI:** 10.1371/journal.pone.0242504

**Published:** 2020-11-24

**Authors:** Michael T. Bixter, Samantha L. McMichael, Cameron J. Bunker, Robert Mark Adelman, Morris A. Okun, Kevin J. Grimm, Oliver Graudejus, Virginia S. Y. Kwan

**Affiliations:** 1 Montclair State University, Montclair, NJ, United States of America; 2 Arizona State University, Tempe, AZ, United States of America; 3 National University of Singapore, Singapore, Singapore; Aalborg University, DENMARK

## Abstract

People encounter intertemporal decisions every day and often engage in behaviors that are not good for their future. One factor that may explain these decisions is the perception of their distal future self. An emerging body of research suggests that individuals vary in how they perceive their future self and many perceive their future self as a different person. The present research aimed to (1) build on and extend Hershfield’s et al. (2011) review of the existing literature and advance the conceptualization of the relationship between the current and future self, (2) extend and develop measures of this relationship, and (3) examine whether and how this relationship predicts intrapsychic and achievement outcomes. The results of the literature review suggested that prior research mostly focused on one or two of the following components: (a) perceived relatedness between the current and future self in terms of similarity and connectedness, (b) vividness in imagining the future self, and (c) degree of positivity felt toward the future self. Additionally, differences in how researchers have labeled the overall construct lead us to propose *future self-identification* as a new label for the three-component construct. Our research built on existing measures to test the validity of a three-component model of future self-identification. Across three samples of first-year undergraduates, this research established the psychometric properties of the measure, and then examined the relationships between the components and four outcome domains of interest: (1) psychological well-being (self-esteem, hope), (2) imagination of the future (visual imagery of future events, perceived temporal distance), (3) self-control, and (4) academic performance. We demonstrated that the three components of future self-identification were correlated but independent factors. Additionally, the three components differed in their unique relationships with the outcome domains, demonstrating the utility of measuring all three components of future self-identification when seeking to predict important psychological and behavioral outcomes.

## Introduction

People encounter intertemporal decisions every day and the choices they make now will impact them far into the future. Unfortunately, people often engage in decisions and behaviors that are not good for their own future. One factor that may explain these decisions is the perception of their distal future self [1]. An emerging body of research suggests that individuals vary in how they perceive their future self, with parallels between how people psychologically treat future selves and other people. For instance, an fMRI study showed that, when thinking about the future self, brain activation in college students closely matched the pattern shown when they thought about a stranger [2], though the precise neural location of this similar processing is debated [3]. As the future self feels more like another person, students may prioritize current rewards (e.g., enjoyment at a party) and disregard potentially large future rewards (e.g., academic success). In other words, intertemporal decisions that favor the present over the distant future may be a result of increased dissociation between the current and future self.

### The present research

Why is there a perceived disassociation between the current and the future self? The present research aims to (1) identify major components of the perceived relationship between the current and future self, (2) extend and develop empirical measures to address this relationship, and (3) investigate whether and how these components uniquely and jointly relate to important outcomes. To address our aims, we conducted a literature review of the psychological research on the perception of the future self. Notably, Hershfield [4] provided a detailed review of the relevant literature. However, since his review in 2011, this body of literature has gained vast momentum. As such, we expand on his review including articles in peer-reviewed journals that (a) were published before January 2019 and (b) empirically measured people’s view of their future self and/or their view of the relationship between the current and future self. The January 2019 cutoff date was used because this is when the literature review was conducted. However, subsequent articles that were published that are particularly relevant for our research aims are still summarized below when appropriate. Our search using PsycInfo and the keyword “future self” yielded 363 articles of which 71 met these review criteria [5–74].

### What makes up perceptions of the future self?

Consistent with Hershfield [4, 75], our literature review shows that most of the research in this area focuses on one or two of the three following components in people’s conception of their future self: (1) the perceived similarity and connectedness between the current and future self, (2) the degree of vividness when the future self is imagined, and (3) the degree of positivity felt toward the future self. It should be noted that researchers have used different labels in referring to these components. Below, we summarize the extant research for each of the three components, discuss the appropriateness of the labels given the items used to assess them, and then suggest novel labels wherever needed to provide clarity in the conceptualization of the construct.

### The relatedness component: Similarity and connectedness to the future self

Twenty-six of the 71 articles in our review included a measure of similarity (and/or connectedness). Much of this research on perceptions of the future self draws on the theoretical work of the philosopher Derek Parfit [76, 77]. According to Parfit [77], the self can be conceptualized as a collection of selves representing the individual at different time points, and sharing a certain degree of psychological similarities and connectedness with one another. This degree of connection and/or similarity between the present and future self is a frequently studied component in recent research on the future self.

Twenty of the 26 articles in our review used a version of the 2-item Future Self-Continuity Measure [21], which was adapted from the Inclusion of Other in the Self Scale [78]. The two items ask about the perceived connectedness and similarity between the present and future self. Participants select one of seven pairs of circles that overlap to an increasing degree, in which one circle represents the current self and the other represents the distal future self. A greater degree of overlap between the two circles indicates greater perceived similarity and connectedness between the current self and the future self. Notably, this measure asks participants to consider their future self at a specific point in time (e.g., 10 years in the future) but does not ask participants to consider the future self in a specific domain (e.g., career, social relationships). In other words, participants express their self-view in a relatively decontextualized (or global) way, thus allowing them to define themselves freely as they see fit. Individuals who score highly on this measure are more likely to engage in future-oriented behavior, such as increased long-term health behaviors [64], greater monetary saving, less-myopic temporal discounting tendencies [21], and decreased acceptance of unethical behaviors [29].

Some researchers have used a modification of the Inclusion of Other in the Self Scale [78] that focuses on the connectedness (i.e., not similarity) to the future self, especially in terms of core identity and psychological characteristics [6, 79–82]. Similarly, research from this perspective finds that connectedness to future selves affects how an individual chooses between rewards that occur at different points in time (i.e., intertemporal choices) [6]. If an individual feels less connected to a self that is more distant in the future, he or she is less likely to adopt behaviors or make choices that would benefit that distant future self (e.g., saving for retirement) and are more likely to engage in behaviors that benefit the present self (e.g., spending all disposable income on short-term consumption). The two labels most commonly used in the literature—similarity and connectedness—do not fully capture both aspects of the component. Therefore, we propose the label “relatedness” which encompasses both critical aspects of similarity and connectedness. According to the Self-Determination Theory [83], relatedness is one of the innate psychological needs critical to self-motivation. In that literature, relatedness entails feelings of connection and similarity with *close others*. These others act as a role model and reinforce self-motivation by representing what the future self could look like. For example, perceived relatedness between students and their role models such as teachers or parents predicts students’ drive for academic achievement [84, 85]. Considering the future self as a special case of the other, we suggest that relatedness represents both perceived connection and similarity between the current and future self. For the remainder of this article, we henceforth use the term relatedness to refer to the component of the future self that represents individual differences in perceived similarity and connectedness.

### The positivity component: Liking and a positive valence towards the future self

Our review found that 35 articles included at least a measure of perceived positivity or negativity of the future self. Akin to the existing literature on the relatedness component, the literature on positive views of the future self has used several different labels. For example, some studies measure “self-prediction positivity” [68], “perceived valence of future time” [44], or “positivity of the future self” [4]. Despite differences in the labels, this component captures the degree to which individuals see their future self as desirable and positive. As such, we agree with Hershfield’s conclusion that the label “Positivity” clearly represents this component [86].

In 13 articles, researchers used a “decontextualized/global” measure of the future-self positivity (i.e., participants indicated how positive they imagine their distal future would be or how much they like their future self in general). The remaining 22 articles included measures of the perceived positivity (or negativity) of future self in terms of specific attributes (e.g., calm, confident, positive, fearful, unhappy) [56, 67] or domains (e.g., future social relationships, leisure activities, finances, and work) [38]. In 15 of these 22 articles, the analyses focused on an overall composite of positivity of future self by averaging participants’ positivity ratings across attributes/domains, or formed separate aggregates for positivity/hoped-for future self and negativity/feared future self [45, 74]. Consistently, previous findings show that positivity of future self predicted greater preparation or beneficial behavior for the future [38] and decreased adolescent delinquency [87, 88].

We identified three articles in which researchers used an alternative to self-report for assessing positivity. In these articles, positive and negative future selves were assessed with a me/not me task in which participants were exposed to positive (e.g., wise, calm, confident) and negative (e.g., dull, fearful, stubborn) adjectives of the future self [55, 65, 67].

### The vividness component: Clarity and ease of imagining the future self

The final component, vividness of the future self, refers to the extent to which individuals conjure a clear image of themselves in the future. In our review of the literature, we found 12 articles in which researchers measured vividness of the future. In the majority of the studies, researchers used self-report measures to assess clarity or vividness of images of the future self [8, 51]. In contrast, two studies used a qualitative approach, coding vividness from participants’ brief descriptions of their future [18, 41]. In terms of the domain-specific versus global focus, seven articles assessed vividness of a global, decontextualized future self [8, 69] and five assessed vividness in specific future domains, including retirement) [20] and best possible self [57]. Examples of domain-specific measures of vividness assess the clarity of specific possible future events (e.g., “the next time you meet a friend”) [89].

Much of the research on the vividness component focuses on manipulating the clarity of future images to experimentally increase the degree of relatedness between the present and future self. For instance, interacting with computer-aged renderings of the self leads individuals to feel more connected between their current and future selves [90], thus suggesting that the extent to which individuals can vividly imagine their life in the future is related to their ability to feel psychologically connected to that future self. This increased level of future self-vividness then leads to behaviors indicative of a more long-term temporal focus, including reduced levels of delinquent behavior [91, 92] and temporal discounting [90]. Similarly, research on episodic future thinking has shown that a higher capacity to imagine events that may occur in the future has a variety of beneficial outcomes. These include increased likelihood of waiting for a larger, delayed reward and more effective emotion regulation skills [89, 93].

Researchers studying vividness have used several labels for this component including “vivid mental imagery” [7], “episodic future thinking” [94], “future clarity” [51], and “vividness of the future self” [4]. Although these programs of research use diverse labels, their primary focus is on measuring the clarity of mental images of the future self. As such, the vividness label proposed by Hershfield [4] clearly captures the essence of this component.

### Labeling the triadic model

Hershfield [4] proposed the *future self-continuity* model to encompass the three components of the future self. However, we suggest that the construct label, future self-continuity, presents issues that might lead to a lack of clarity in this literature. Below, we (1) discuss a theoretical issue associated with the use of future self-continuity, (2) articulate why this label does not fully reflect the proposed construct, and (3) provide an alternative label—future self-identification.

Hershfield’s “future self-continuity” label is derived from Parfit’s [76, 77] concept of continuity between temporal selves. According to Parfit, continuity is the overall strength of overlapping links between all sequential pairs of selves. Contrastingly, the three components in Hershfield’s [4] model and the extant literature concern the direct relation between the current and a future self at a given point in time. In other words, the three components only include perceptions of the relatedness, vividness, and positivity of *a particular* future self. Consequently, an individual may feel highly related, positive, and vivid about a particular future self but may or may not feel a high level of continuity between his or her entire chain of temporal selves.

Additionally, the future self-continuity label focuses exclusively on relatedness between temporal selves, but overlooks the positivity and vividness components. While Parfit [77] discussed vividness and positivity as elements of the future self, they are not necessary to his notion of continuity. Under his conceptualization of continuity, the current and future self may be perceived as highly psychologically continuous without a highly positive or vivid view of the self in the future. An individual with chronic pain may feel highly connected or continuous with a negative future self. Similarly, upon being laid off from a job, a person may fail to see a clear vision of what the future holds despite a strong sense of connection to that future self. This distinction is important because the three components are conceptualized as related but distinct [4]. Consequently, the existing label fails to reflect distinctions in the valence and visualization of the future and does not lend itself to representing all three proposed components under one unified construct.

In light of these issues, we suggest a new label—*future self-identification*—to represent the overall construct that includes the three components (relatedness, vividness, and positivity). According to the American Psychological Association [95], “identification is the process of associating the self closely with other individuals.” The strength of identification plays an important role in decision-making, as those with higher identification are more likely to consider the shared connection and values with those they identify with and less likely to act based on self-interest [96]. Previous research found that individuals who indicated higher group identification were more likely to stay committed to the group and less likely to leave and join another [97]. Individuals were more likely to contribute to a failing group only when group identification was salient as opposed to personal identification [98].

Although targets of identification are generally others or groups, we suggest the process of identification applies to the perception of the future self. This aligns with the analogy of the “future-self-as-other”. Of course, a future self is still physically tied to a current self, but psychologically there are parallels between how people treat future selves and how they treat other people. Research utilizing fMRI techniques found that making decisions about a distal future self did not elicit the same activation in the medial prefrontal cortex and rostral anterior cingulate as did making decisions about the current self [2]. These cortical midline structures are involved in the processing of self versus other information [99], though there is debate about the precise neural location of this similar processing [3]. The difference in activation between these brain regions for current-self-related and future-self-related decisions also predicted subsequent measures of temporal discounting [2]. Recently, Molouki and Bartels [100] found that there were parallels between how people treat future selves and others, such as similar factors underlying the allocation of resources to both future selves and other individuals. However, this research also showed that people generally allocate more resources to future selves than they allocate to others, suggesting that future selves are not treated exactly like other people.

We propose that people who feel more related to the future self, have more positive feelings towards it, and can imagine it more clearly, will more strongly identify with the future self than those who do not. These elements closely align with the components that impact identification within the group identification literature. Specifically, Henry, Arrow, and Carini [101] argued that group identification will be stronger in groups with greater intra-individual similarity [102, 103], more positive feelings towards group members, and sharing more vivid goals [104, 105]. Given the parallel between the process of identification and the perceived relation between the current and future self, we suggest a departure from previous research by assigning a new label—*future self-identification*—as the overarching construct encompassing the three most common components of future self in the literature. Together, the three components of future self-identification tap into the extent to which an individual relates their current self to their future self, how clearly they envision their future self, and how much they like their future self.

### Relationships among the three components

At the time of our review, no research had empirically examined the relationships among the three components of future self-identification and few researchers even examined the relation between two of the components in one study. However, results of the existing research suggest positive relationships among the three components. For example, one study found positive associations between measures of liking the future self and measures of feeling similar to the future self, suggesting that the *positivity* and *relatedness* components may be positively related [21]. This finding is consistent with research on Temporal Self-Appraisal Theory showing that individuals often report feeling closer to favorable than unfavorable future selves [73, 106, 107]. Moreover, a recent study found that experimentally manipulating the valence of expected change in personal characteristics (e.g., personality, preferences, morality, experiences) affected perceived continuity between current and future selves [108]. Specifically, positive changes in the future produced higher future self-continuity, whereas negative changes were particularly disruptive to perceived self-continuity with future selves. These results further suggest a relationship between the valence of perceived future selves and degrees of relatedness.

Considering the relationship between *relatedness* and *vividness*, experimental studies show that exposure to computer-aged images (i.e., a manipulation that presumably induced vividness of the future self) enhanced perceived relatedness to the future self [40, 90]. Additionally, individuals who could vividly imagine their future self were more likely to relate to the future self, suggesting that the vividness and relatedness components may be positively related [8]. This recent study also found that individuals higher in future self-vividness experienced steeper increases in relatedness to the future self over time. However, another experimental study did not find a significant relationship between vividness and relatedness [49]. This research reported that, after writing about their possible future selves, participants’ ratings of vividness of their future selves did not predict ratings of the similarity of their future self. Although vividness of future self ratings was a negative predictor of number of undesirable words generated for the future self, they did not predict the number of desirable words for the future self [49]. Thus, the findings from studies of the relationship between vividness and positivity are inconclusive.

Another notable observation of our review is that the majority of studies on the validity of future self-identification focused on predicting *temporal discounting* [2, 6, 29], and with few exceptions, other important outcomes have received less attention. For instance, only three studies examined *achievement* outcomes. These studies found that relatedness to future self positively predicted college grade point averages and negatively predicted procrastination [5, 7], and positivity of future self predicted higher academic motivation [55].

Further, several studies examined one of the components of future self-identification and its relations with *intrapsychic* outcomes. These studies found that (a) *relatedness* predicted more positive and less negative affect [7], more psychological well-being [14], and less depression [67]; (b) *positivity* predicted less anxiety and depression [19, 31, 67], more life satisfaction and state self-esteem [33, 39], and higher quality of life [64]; and (c) *vividness* predicted higher life satisfaction, greater positive affect, and less negative affect and depression [49].

A gap in previous research involves testing the independent predictive validity of the three components. One exception examined two of the components, finding that vividness, but not relatedness, predicted more positive and less negative affect [51]. Given the positive relationships that have been observed among the three components, it is important to test the independent predictive validity of each component.

Since the time of our literature review, Sokol and Serper [109] tested a measure of all three components within a single framework. A number of interesting findings emerged from this research. First, the results of their confirmatory factor analysis show that the three components are positively correlated ranging from .46 to .59 and they are independent factors, supporting a triadic model of this construct. Second, each component was significantly correlated with one or more indicators of less temporal discounting (e.g., consideration of future and immediate consequences) and intrapsychic adjustment (e.g., life satisfaction and hopelessness). Third, comparisons of the strength of the correlations between each component suggest that positivity was the strongest predictor of consideration of the future consequences. However, the three components did not differ in terms of their correlations with a temporal discounting task.

We acknowledge the important contribution of this work. However, two crucial empirical issues remain. First, although this study examined all three components, it did not report the extent to which the three components *independently* predict outcome variables of interest. This is important because each component may show different patterns of unique relationships with outcomes depending on how much overlap it shares with the other components. This is a crucial issue as researchers interested in future self-identification may wish to understand the unique contribution of each component for different outcomes (e.g., for the design of experimental manipulation/intervention). Second, the convergent, divergent, and independent predictive validity of the frequently used “global and decontextualized*”* measures of the future self remain unknown. In Sokol and Serper’s [109] assessment of the three components, participants considered the domains of their future self chosen by the researchers (e.g., values, personality, beliefs, appearance, and family relations). However, much of the literature on this topic assessed the components with global, decontextualized measures, emphasizing participants’ freedom to consider the aspects of the future self that are most important to them. Accordingly, we consider these issues in the present research.

### Overview of the present research

Our review of the literature supports the idea that there are three major components of future self-identification that have downstream consequences. In the present research, we will address three major research goals: (1) advance the conceptualization of the relationship between the current and future self, (2) test a measurement model of the factor structure of future self-identification by extending and developing items tapping into the three components of future self-identification, and (3) examine whether and how the components of future self-identification uniquely relate to intrapsychic and achievement outcomes. To study all three components in one study, we aim to minimize methodological differences in the measures by framing all items in terms of the decontextualized, global future self. Our literature review shows that few studies on this topic have examined the relationships between multiple components of future self-identification and outcomes related to intrapsychic adjustment and objective markers of achievement. Therefore, the present research focuses on the following four outcome domains to help clarify the unique relationships with the three components of future self-identification: (1) psychological well-being (self-esteem and state hope), (2) imagination of the future (visual imagery of future events and perceived temporal distance), (3) self-control, and (4) academic performance.

We organize the report below into four parts. It was first necessary to extend and develop items that tap into all three components of future self-identification. In Part I, we examine whether the future self-identification items have a triadic factor structure. In Part II, we further test the reliability of the future self-identification factors by assessing test-retest reliability and the replicability of the factor structure in a new sample. In Parts III and IV, we then ascertain the unique relationships between the three components of future self-identification and our outcomes of interest. Because prior research often only included measurement of one component of future self-identification, Parts III and IV will help clarify the independent relationships between the three components and important intrapsychic and achievement outcomes.

A crucial aspect of this research is the life stage of our participants. We chose participants going through a major age-normative transition—starting college—because such transitions are junctures in people’s lives. Although older students (e.g., third-year college students) may have more information in predicting their senior year or beyond, we expect that differences in how entering college students conceive of their future may shape important outcomes such as academic performance. As many students drop out of college after the first year [110], research targeting entering freshmen such as the present one may give insight into early interventions to increase retention. Finally, our future self-identification scale used a future self in 10 years as a reference point. Five or six years after graduation (approximately 10 years from starting college) is when many critical life decisions are made (e.g., career choice, marriage, and family decisions). Degrees of identification with a future self at this time frame may be impactful on academic outcomes during college.

### Part I: Factor structure of future self-identification items

In Part I, we used confirmatory factor analysis to test whether the extended and newly developed items of the three components of future self-identification represent discriminant factors. Specifically, we tested whether the best fit to the future self-identification items was provided by a three-factor model. As described above, relatedness, vividness, and positivity were expected to be separate components of perceived psychological identification with the future self. Given that each of the three components focuses on different aspects of the future self (e.g., degree of imagination, valence), we predicted that the three components would be discriminant (but positively correlated) factors within an overall model.

### Method

#### Sample

The current research drew on a sample from an ongoing longitudinal study examining factors that promote academic motivation and performance among college students. The data reported here have not been reported elsewhere. Participants were first-year college students enrolled in Introduction to Psychology or General Chemistry courses at a large public university in the United States. All participants were permanent residents or citizens of the U.S. and were 18 years or older at the beginning of the study. Five hundred forty-nine undergraduate students participated in the study. The sample was 55.9% (*n* = 307) female. The racial and ethnic breakdown of the sample was as follows: 56.3% Caucasian (*n* = 309), 20.4% Hispanic or Latino (*n* = 112), 13.2% Asian (*n* = 72), 3.3% African American (*n* = 18), 3.1% Middle Eastern (*n* = 17), 0.9% Native American (*n* = 5), 2.2% Other (*n* = 12), and 0.7% unreported (*n* = 4).

#### Measures

*Future self-identification*. Participants completed six items that were adapted or newly developed to tap into the three proposed components of future self- identification: two items designed to assess relatedness between the current and future self, two items designed to pertain to vividness of the future self, and two items designed to relate to positivity toward the future self. All items asked participants to think of themselves approximately 10 years in the future (i.e., five years following college graduation). We describe these items below and will discuss their psychometric properties in the Results section.

The two relatedness items were adapted from the literature [21] and are based on the Inclusion of Other in the Self Scale [78]. Participants were first presented with seven pairs of increasingly overlapping Euler circles. The two circles represent a participant’s current and future self. The first item asks participants to select the pair of circles which best describes how *similar* they feel to their future self (1 = *not at all similar to my future self*, 7 = *very similar to my future self*). The second item asks participants to indicate the pair of circles which best describes how *connected* they feel to their future self (1 = *not at all connected to my future self*, 7 = *very connected to my future self*).

To assess the vividness component, we developed two items that assessed: (1) individual differences in the tendency to create a *clear* mental image when imaging the future self and (2) the *ease* with which individuals can create mental imagery of their future selves. For the *clarity* item, participants were asked “When you imagine your future self, how vividly do you picture it?” (1 = *not at all vividly; I do not have a clear image in my head of my future self*, 7 = *very vividly; I have a very clear image in my head of my future self*). For the *ease of visualization* item, participants were asked “How easy is it for you to visualize a mental picture of your future self?” (1 = *Very difficult*, 7 = *Very easy*).

The two positivity items assessed the extent to which participants like and hold a positive attitude toward their future selves. We adapted one item from Ersner-Hershfield et al. [21] that measured the extent to which participants *like* their future selves: “How much do you like your future self five years after graduating from college?” (1 = *don't like at all*, 7 = *like as much as possible*). We also included a new *valence* item that had participants directly rate the degree of negativity/positivity felt towards their future self. Participants responded to the prompt, “When I think about the future, my future self feels. . .”, with participants using a slider to indicate the degree of positivity (0 = *Very Negative*, 100 = *Very Positive*). Later waves in the longitudinal study also included a seven-point valence item. We found similar factor analytic results whether the seven-point or 100-point valence item was used in the analyses.

#### Procedure

We administered surveys online via Qualtrics software. Surveys were typically completed in 20 to 30 minutes. Participants received $10 for completing the survey. Data collection occurred approximately one week into the fall semester of participants’ first year in college. All study materials and data collection procedures were approved by the Institutional Review Board at Arizona State University. Participants in all samples provided informed consent prior to completing the surveys.

#### Analysis

**Fig 1:**
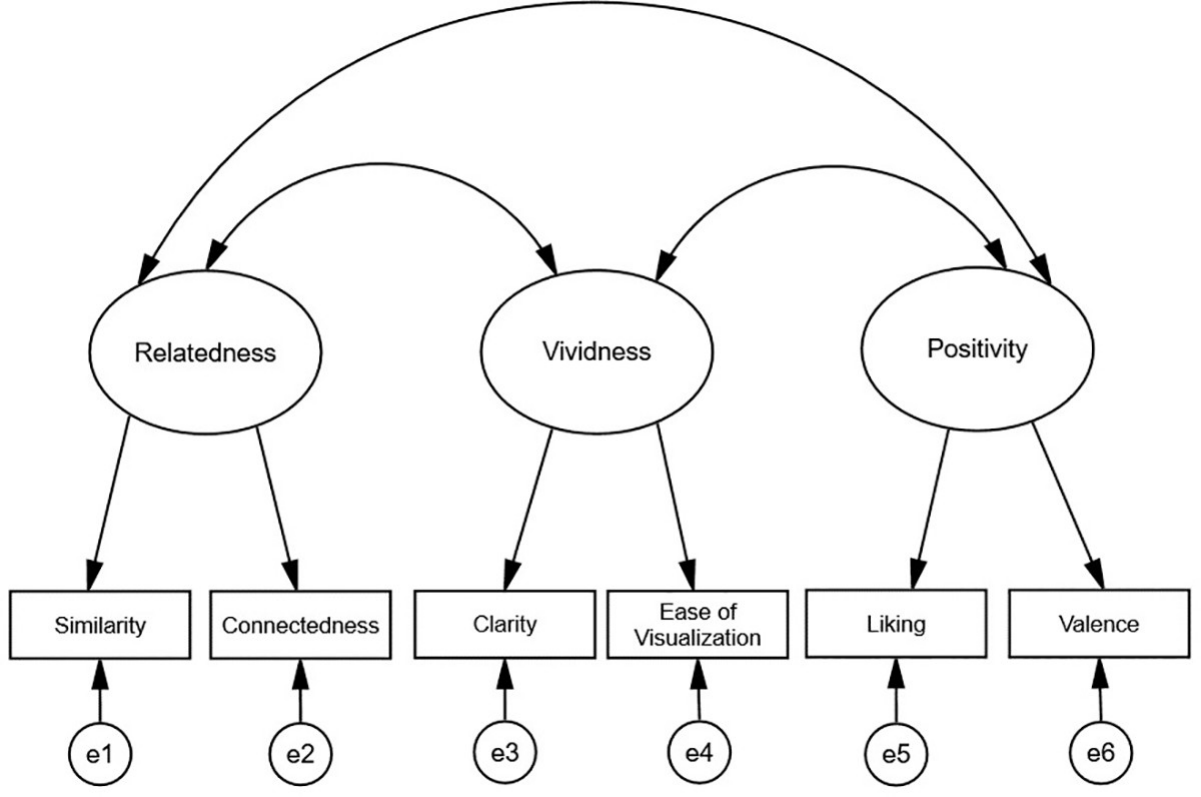
The three-factor model of future self-identification. See the Method section in Part I for a description of the future self-identification items. The Method section in Part IV includes description of the revised future self-identification items.

We used confirmatory factor analysis to examine the factor structure of the future self- identification items. Five models were constructed to test competing conceptualizations of the future self-identification construct. In the one-factor model, all six future self-identification items loaded onto a single latent variable. A three-factor model was fit where the two relatedness items were indicators of the first factor, the two vividness items were indicators of the second factor, and the two positivity items were indicators of the third factor (see Fig 1 for a visual representation of the three-factor model). For completeness, we also tested all of the possible two-factor models: (a) relatedness items were loaded on one factor and positivity/vividness items were loaded on a second factor, (b) vividness items were loaded on one factor and relatedness/positivity items were loaded on a second factor, and (c) positivity items were loaded on one factor and relatedness/vividness items were loaded on a second factor.

Across all five models, we implemented the following specifications: (a) factor means and variances were set to zero and one, respectively; (b) the loadings for the unique factors were set to one; (c) the means of the unique factors were set to zero; (d) the factor loadings, the intercepts of the indicators, and the variances of the unique factors were freely estimated. For the multi-factor models (i.e., the two-factor and three-factor models), covariances between the latent variables were also freely estimated.

### Results

Table 1 shows the descriptive statistics for the six future self-identification items. The fit statistics of the five confirmatory factor analyses are presented in Table 2. Fit statistics included the chi-square test, the comparative fit index (CFI), the root mean square error of approximation (RMSEA) with 90% confidence intervals, and the standardized root mean square residual (SRMR). Due to the chi-square test being influenced by sample size, the CFI, RMSEA, and SRMR were also used as the indices to examine model fit in the study. Per general conventions, CFI values above .90, RMSEA below .08, and SRMR below .08 were used as indications of reasonable model fit [111, 112].

**Table 1:** Descriptive statistics of the future self-identification items.

Item	Sample 1		Sample 2	Sample 3
	Time 1	Time 2		
Similarity	4.09 (1.27)	4.26 (1.24)	4.10 (1.35)	4.18 (1.42)
Connectedness	4.22 (1.57)	4.27 (1.47)	4.14 (1.51)	4.48 (1.55)
Clarity	4.61 (1.59)	4.69 (1.49)	4.54 (1.58)	4.74 (1.46)
Ease of Visualization	4.71 (1.53)	4.66 (1.57)	4.53 (1.53)	4.83 (1.44)
Liking	5.89 (1.05)	5.70 (1.11)	5.79 (1.14)	5.62 (1.20)
Valence	78.82 (15.67)	79.37 (16.33)	76.49 (17.05)	6.11 (0.94)

*Notes*. Means (and standard deviations) are included in the table. See the Method section for a description of the future self-identification items. Sample 1 is described in Part I, Sample 2 is described in Part II, and Sample 3 is described in Part IV. The valence item for Samples 1 and 2 was on a 100-point scale, and Sample 3 was on a 1 to 7 scale.

**Table 2:** Fit statistics of the five confirmatory factor analyses in Part I.

Model	χ^2^ [df, sig.]	CFI	RMSEA [CI]	SRMR
One Factor	174.37 [9, < .001]	.839	.183 [.160, .207]	.089
Two Factor: R and P-V	92.71 [8, < .001]	.918	.139 [.114, .165]	.063
Two Factor: V and R-P	84.35 [8, < .001]	.926	.132 [.107, .158]	.060
Two Factor: P and R-V	99.02 [8, < .001]	.911	.144 [.119, .170]	.073
Three Factor	5.49 [6, .482]	1.000	.000 [.000, .053]	.012

*Notes*. CFI = comparative fit index; RMSEA = root mean square error of approximation; SRMR = standardized root mean square residual. One Factor Model: all six future self-identification items were loaded onto a single latent variable. Two Factor Models: (a) R and P-V: relatedness items were loaded on one factor and positivity-vividness items were loaded on a second factor (b) V and R-P: vividness items were loaded on one factor and relatedness/positivity items were loaded on a second factor, and (c) P and R-V: positivity items were loaded on one factor and relatedness-vividness items were loaded on a second factor. Three Factor Model: the two relatedness items were indicators of the first factor, the two vividness items were indicators of the second factor, and the two positivity items were indicators of the third factor.

The one-factor model and the three two-factor models did not reach acceptable levels of fit on any of the indices (e.g., RMSEAs > .13), suggesting these models did not adequately account for the covariance structure of the data. The fit of the three-factor model was good and reached acceptable levels on all fit indices. One common method to assess whether one model provides a better fit to the data than another model is if the change in CFI (ΔCFI) is more than .01 [113]. Using this criterion, the three-factor model provided a better fit to the data than the one-factor model and the three two-factor models.

Table 3 contains the standardized factor loadings. In the three-factor model, all items loaded significantly (*p*s < .001) on their designated factors. The correlations among the three factors were all positive and significant: relatedness and vividness (*r* = .38, *p* < .001), relatedness and positivity (*r* = .38, *p* < .001), vividness and positivity (*r* = .61, *p* < .001). These results support a three-factor structure of the future self-identification items.

**Table 3:** Standardized factor loadings of the three-factor model of future self-identification.

	Sample 1	Sample 2	Sample 3
Item Factor	Time 1		
Similarity ← Relatedness	.415	.626	.649
Connectedness ← Relatedness	.968	.721	.830
Clarity ← Vividness	.853	.900	.903
Ease of Visualization ← Vividness	.944	.906	.864
Liking ← Positivity	.662	.618	.805
Valence ← Positivity	.726	.751	.726

*Notes*. All factor loadings were statistically significant (*p*s < .001). See the Method section (Parts I and IV) for a description of the future self-identification items. Sample 1 is described in Part I, Sample 2 is described in Part II, and Sample 3 is described in Part IV.

#### Convergent validity

Internal convergent validity tests the extent to which factors are well measured by their respective indicators. The Fornell-Larcker criterion for convergent validity [114] requires the average variance extracted for each factor to be approximately 0.5 or above. Average variance extracted refers to the average variance of indicators that is captured by the latent factor in relation to the amount of variance of the indicators that is unique. This is measured by taking the average of each factor’s squared standardized factor loadings. The average variance extracted for the relatedness (0.55) and the vividness (0.81) factors were above 0.5, with the average variance extracted for the positivity factor (0.48) being just slightly below 0.5. The lower average variance extracted for the positivity factor could be due to the items being assessed on different response scales. Data from Part IV below that used the same response scale for the items produced an average variance extracted for the positivity factor of 0.59.

#### Discriminant validity

Internal discriminant validity tests the extent to which factors are independent of one another within an overall model. The Fornell-Larcker criterion for discriminant validity [114] requires the average variance extracted of a latent variable to be greater than the squared correlations between the latent variable and other factors in the model. The squared correlations between the three factors were: relatedness and vividness (.14), relatedness and positivity (.15), and vividness and positivity (.37). As reported above, the average variance extracted of the three factors were all greater than these squared correlations (0.55 for relatedness, .81 for vividness, and .48 for positivity), suggesting good discriminant validity of the three-factor model.

## Part II: Test-retest reliability and replicability of the three-factor model

In Part II, we focus on examining the reliability and replicability of the three-factor structure of the future self-identification items. Specifically, we assess (1) test-retest reliability and (2) the replicability of the model in a second sample of participants.

### Method

#### Samples

Sample 1 contained the same group of participants included in Part I. For the test-retest reliability analyses reported below, two waves of data were used. Time 1 was the same data collection reported in Part 1 and occurred approximately one week into participants’ first year in college. Time 2 data collection occurred approximately five weeks after Time 1 (i.e., approximately six weeks into participants’ first year in college). Three hundred and sixty-nine participants (67%) from Time 1 returned and completed the future self-identification items in Time 2.

Sample 2 was a different group of 765 undergraduate students who completed the future self-identification items. All participants were permanent residents or citizens of the U.S. and were 18 years or older at the beginning of the study. Of these students, 55.6% (*n* = 425) were female. The racial and ethnic breakdown of the sample was as follows: 54.0% Caucasian (*n* = 413), 19.9% Hispanic or Latino (*n* = 152), 14.5% Asian (*n* = 111), 3.9% African American (*n* = 30), 2.8% Middle Eastern (*n* = 21), 1.2% Native American (*n* = 9), 2.4% Other (*n* = 18), and 1.4% unreported (*n* = 11).

#### Measures and procedure

The same procedure and future self-identification items from Part I were used in Part II.

### Results

#### Test-retest reliability of the three-factor model

We first report the test-retest reliability of the three-component model of future self-identification. The test-retest reliability analyses reported below utilized data from two time periods separated by approximately five weeks (Time 1 and Time 2). The three-factor model from Part I (see Fig 1) was fit to the Time 1 and Time 2 data simultaneously. This produced six latent factors (the relatedness, vividness, and positivity factors at both Time 1 and Time 2). We implemented the same specifications as was done in Part I, with the following additional specifications: (a) all six latent factors were allowed to covary; (b) unique factors were allowed to covary across time periods (e.g., the unique factor for the relatedness item at Time 1 with the unique factor for the relatedness item at Time 2).

*Factorial invariance testing*. Prior to measuring the test-retest reliabilities of the future self-identification factors, we first established factorial invariance across the two time periods. This ensured that the latent variables (e.g., the relatedness, vividness, and positivity factors) measured the same constructs at the two time periods. To test for factorial invariance, we followed the steps outlined by Widaman, Ferrer, and Conger [115]. Specifically, we ran three models with increasingly strict constraints placed on the estimated parameters: (1) *configural invariance*, where the same factor structure is fit at the two time periods but all free parameters are estimated separately across time; (2) *weak factorial invariance*, where factor loadings are constrained to be equal across time; and (3) *strong factorial invariance*, where factor loadings and item intercepts are constrained to be equal across time.

If adding constraints to the model leads to a significant change in model fit, factorial invariance would not be achieved. The CFI model fits were good for all three models: the configural invariant model (.991), the weak factorial invariant model (.989), and the strong factorial invariant model (.977). Moreover, ΔCFI between the configural and weak factorial invariant models (.002) and between the weak and strong factorial invariant models (.012) did not or slightly exceeded .01. As a result, we concluded that strong factorial invariance was established for the three-factor model across the two time periods.

*Test-retest results*. The overall fit of the strong factorial invariant model reached acceptable levels on the fit indices, χ^2^(45) = 98.74, *p* < .001; CFI = .977; RMSEA = .057 [.042, .072]; SRMR = .038. The test-retest reliabilities of the three factors were as follows (see Table 4): relatedness (*r* = .65, *p* < .001), vividness (*r* = .80, *p* < .001), and positivity (*r* = .85, *p* < .001). These significant correlations demonstrate the stability of individual differences in the future self-identification components over the five-week period between Time 1 and Time 2.

**Table 4:** Correlations between the three factors of future self-identification at time 1 and time 2 in Part II.

	Relatedness1	Vividness1	Positivity1	Relatedness2	Vividness2	Positivity2
Relatedness1	--					
Vividness1	.48	--				
Positivity1	.54	.63	--			
Relatednes2	**.65**	.43	.46	--		
Vividness 2	.47	**.80**	.55	.57	--	
Positivity2	.50	.59	**.85**	.57	.69	--

*Notes*. All correlations were statistically significant (*p*s < .001). Correlation coefficients in bold are test-retest reliabilities.

#### Replicability of the three-factor model

We replicated the three-factor model in a second sample of first-year students (for descriptive statistics, see Table 1). The same three-factor model used in Part I was fit to the Sample 2 data. See Table 3 for the standardized factor loadings. The fit of the model reached acceptable levels of fit, χ^2^(6) = 25.71, *p* < .001; CFI = .987; RMSEA = .066 [.041, .093]; SRMR = .024, demonstrating the validation of the three-factor model across multiple samples. Moreover, the three-factor model significantly improved model fit over the one-factor and all two-factor models (ΔCFIs > .06).

## Part III: Future self-identification and psychological resources and outcomes

Having established the reliability and internal validity of the measurement of the three components of future self-identification, we tested research hypotheses and questions pertaining to the external relationships between the three components and outcome variables of interest. In Part III, we focused on the following four domains: (1) psychological well-being, (2) visual imagery of future events, (3) self-control, and (4) academic performance. Prior research has linked future self-relatedness to all of these domains. However, by almost exclusively focusing on the relatedness component, the extent these relationships are driven by relatedness *per se* remains unclear. As demonstrated in Parts I and II, the three components of future self-identification are positively correlated. This implies that prior research showing relationships between relatedness to the future self and other variables might have been driven by shared variance with vividness or positivity. The dependent variables included in Part III, therefore, provide a critical test of whether future self-identification involves three distinct psychological components that exhibit similar or different patterns of concurrent and predictive relationships. If future self-identification is best represented as a superordinate, unitary construct, we would expect all three components of future self-identification to exhibit similar patterns in their relation to these four domains. In contrast, if the factors are distinct, we would expect more discrimination in the association between the components of identification and the outcomes of interest.

Our goal was therefore to clarify the independent influences of the three components. We strategically chose our outcome variables to achieve this goal. For example, we chose the psychological well-being outcomes because they are conceptually more similar to the positivity component than the other two components of future self-identification. As a result, by including measurement of all three components within a single study, we were able to test if there are unique relationships with the other two components after adjusting for the relationship with the positivity component. Similarly, we included the visual imagery variables because they are more similar to the vividness component than the other two, but it remained an empirical question if there exist unique relationships with the other two components after adjusting for the vividness component. For the other two outcome domains (self-control and academic performance), there is less of a clear conceptual overlap between the construct and one of the future self-identification components. Because prior research that investigated the relationship between future self-identification and these two domains only included the relatedness component, the unique association between the vividness and positivity components with both self-control and academic performance remain empirical questions. Any distinctive relationships observed between the future self-identification components and outcomes of interest will demonstrate the utility of adopting a three-component model of the construct.

### Psychological well-being

The relatedness component is positively related to satisfaction with life [116]. Perceiving greater relatedness between current and future selves appears to confer benefits to present psychological and subjective well-being. Additionally, relatedness of the future self is positively related to positive affect and negatively related to negative affect [7]. Relative to individuals who perceive less relatedness between selves over time, those who perceive greater relatedness appear to spend more time in positive affective states (e.g., happy, joyful, and fun times) and less time in negative states (e.g., depressed, unhappy, and frustrated).

By measuring all three components of future self-identification, we were able to test their unique relationships with psychological well-being adjusting for the other components. In Part III, self-esteem was chosen as the psychological well-being variable of interest. Due to the conceptual similarity between the future self-positivity items and self-esteem, we expected that the positivity component would be the strongest predictor of self-esteem. Because the positivity component relates specifically to attitudes toward one’s future self, we modified the Rosenberg Self-Esteem Scale [117] with individuals’ future selves as the reference. This allowed us to assess the unique effects of the three future self-identification components on both current and future self-esteem. We predicted a stronger relationship between the positivity component and future self-esteem as compared to current self-esteem. These predictions highlight the idea that individuals can have different evaluations of self-worth and personal value (i.e., self-esteem) for selves at different time periods (e.g., past, current, and future selves). Moreover, it remained an empirical question if relatedness and vividness demonstrate independent relationships with current and future self-esteem after adjusting for positivity.

### Visual imagery of future events

Self-reported ability to create vivid mental images is positively associated with future-oriented behavior, such as reduced levels of procrastination [7]. Individuals may also feel more psychologically connected to a future self if they can more easily visualize details associated with events surrounding that future self. In one study [8], college students at the beginning of an academic semester were measured on how vividly they could imagine aspects of their future self at the end of the semester. Ratings of vivid imagery were positively related to perceived relatedness between the current self and the future self at the end of the academic semester.

To measure visual imagery of future events in the current study, we chose events surrounding two future time periods: (1) vividness of college graduation, and (2) vividness of a typical work week five years after college. These future time periods were chosen because events surrounding college graduation and five years post-graduation are relevant for the long-term goals students possess throughout college. Because visual imagery of future events relies upon the extent to which one holds a vivid conception of the future, we predicted that the vividness component of future self-identification would have the strongest relationship with the visual imagery outcomes. As the current study included all three future self-identification components within a single model, we were able to test if relatedness and positivity demonstrate unique relationships with visual imagery of future events after adjusting for future self-vividness.

### Self-control

Resisting the temptation of immediate gratification in order to maximize long-term rewards typically requires a degree of self-control [118–122]. High future self-identification may promote self-control. If people feel strongly identified with their future selves, they may be better able to eschew behaviors that lead to short-term gratification but negatively impact long-term well-being. In past research, the relatedness component of future self-identification correlated positively with a measure of self-control [5, 29]. Individuals high in future self-identification may be better able to shift their temporal focus to the future instead of overly weighing present priorities. In fact, greater perceived relatedness to the future self is positively related to consideration of future consequences and negatively related to consideration of immediate consequences [5].

Prior research only studied the relationship between the relatedness component of future self-identification and self-control. Consequently, it remains unclear which component(s) of future self-identification predict self-control. By including all three components within a single study, the present investigation helps clarify the nature of the relationship between future self-identification and self-control.

### Academic performance

Lastly, we examined the relationship between future self-identification and academic performance. Academic achievement often requires the eschewing of immediate gratification (e.g., studying instead of going out with friends) in the service of long-term benefits (e.g., higher grade point average). Similar to the relationship between future self-identification and self-control described above, future self-identification may promote academic achievement by regulating behaviors to be more aligned with long-term goals. Evidence of this includes the negative relationship between perceived relatedness with future selves and academic procrastination [7]. Individuals with a greater perceived overlap between current and future selves appear to be less likely to delay completion of academic tasks.

As it relates to academic performance, a significant relationship was observed between the relatedness component of future self-identification and college grade point average [5]. Individuals with greater perceived relatedness between current and future selves at the beginning of an academic semester have higher grade point averages at the end of the semester. However, it remains an open question whether vividness and positivity would also be significant predictors of subsequent academic performance. The inclusion of all three components of future self-identification within a single study allows us to examine this research question. Moreover, we were also able to test the indirect effects of the three components on academic performance through self-control. This indirect effect was observed for relatedness by Adelman et al. [5] but further testing whether indirect effects are observed for the other two components may help clarify the possible mechanisms that underlie the relationship between future self-identification and academic outcomes.

### Method

#### Samples

In Part III, we used the same two samples that were used in Part II.

#### Measures

*Future self-identification*. We used the same future self-identification items as described above.

*Current self-esteem*. We used the Rosenberg Self-Esteem Scale [117] to measure participants’ current self-esteem. The scale consists of 10 items that measure global self-esteem (e.g., “On the whole, I am satisfied with myself”). All items are answered on a seven-point scale (1 = *Strongly disagree*, 7 = *Strongly agree*). The internal consistency of the Rosenberg Self-Esteem Scale was excellent (Cronbach’s α = .900).

*Future self-esteem*. We created a modified version of the Rosenberg Self-Esteem Scale with individuals’ future selves as the reference. The following prompt was given to participants: “Imagine the person you will become five years after you graduate from college.” This future reference was then used for all items, which were modified from the original scale to deal with the future self instead of the present self (the modified item: “On the whole, I *will be* satisfied with myself”). Eight of the ten items from the original scale were deemed appropriate to be adapted for a future self. The two items excluded were: “I certainly feel useless at times” and “I wish I could have more respect for myself.” These items were excluded from the future self-esteem scale because they would have required a significant change to the original item to be applicable to the future self. All items on the future self-esteem scale were answered on a five-point scale (1 = *Strongly Disagree*, 5 = *Strongly Agree*). The internal consistency of the scale was good (Cronbach’s α = .835).

*Visual imagery of future events*. We created a modified version of the Vividness of Visual Imagery Questionnaire (VVIQ) [123] to measure visual imagery of future events. Three items were related to events around college graduation (VVIQ Graduation), and three items pertained to events around five years after graduation (VVIQ Post-Graduation). An example item from VVIQ Graduation was “Hearing my name called and walking across the stage.” An example item from VVIQ Post-Graduation was “Going home at the end of the day and walking through the front door.” All items were answered on a seven-point scale (1 = *No image at all*, *you only "know" that you are thinking of the situation*, 7 = *Perfectly clear and as vivid as normal vision*). The internal consistency was acceptable for both VVIQ Graduation (Cronbach’s α = .883) and for VVIQ Post-Graduation (Cronbach’s α = .775).

*Self-control*. We used the 10-item Brief Self-Control Scale [124] which was designed to measure individual differences in self-control. Example items from the scale included: “I have a hard time breaking habits”, “Sometimes I can’t stop myself from doing something, even if I know it is wrong”, and “I often act without thinking through all the alternatives.” All items were answered on a five-point scale (1 = *not at all like me*, 5 = *very much like me*). The internal consistency of the scale was acceptable (Cronbach’s α = .797).

*Academic performance*. We used an objective measure of academic performance, namely the cumulative grade point average after the second semester at the university. The maximum grade point average was a 4.0. Participants consented to grant us access to their official academic records through the Institutional Analysis Office of the university.

#### Procedure

Data were collected from participants approximately one week into the fall semester of their first year in college. The analyses dealing with visual imagery of future events, self-control, and academic performance were carried out on data from Sample 1 (see Parts I and II for a description). The analyses dealing with self-esteem used data from Sample 2 (see Part II for a description). Participants in Sample 1 did not complete both the current and future self-esteem variables during the same wave of data collection. Because the focus of the present study is on concurrent relationships between future self-identification and self-esteem, the analyses that contain the self-esteem variables used data from Sample 2. Participants in Sample 2 also completed the self-control and visual imagery of future events measures. The patterns of correlations between the three future self-identification components and these dependent variables for Sample 2 were the same as those for Sample 1. Specifically, *z* tests demonstrated that none of the correlation coefficients between the three components and these dependent variables showed significant differences between Samples 1 and 2. These results are available upon request.

### Results

Given the differences in scale formats for the future self-identification items, we first standardized the items and averaged the two items separately for each of the three components. We then used participants’ scores on these three components to predict the outcome variables of interest. We report both the zero-order correlations and multiple regression coefficients. The latter analyses were to test the unique relationships between the three future self-identification components and the dependent variables. Finally, a path analytic model is included at the end of the Results section to test the indirect effects of the future self-identification components on academic performance through self-control. For all regression models, multicollinearity was not an issue with all variance inflation factors being less than two.

#### Self-esteem

We examined the zero-order correlations of the three future self-identification components with the dependent variables (Tables 5–8). For the self-esteem variables, the correlations were positive and significant for all three components (ranging from .25 to .64), with the strongest correlations being observed for positivity. For both current and future self-esteem, the correlation with positivity was significantly higher than with relatedness and vividness (*z*s > 3.81, *p*s < .001).

**Table 5:** Multiple regression analyses of future self-identification components predicting psychological well-being.

	Part III			Part IV		
	Zero-order correlation (*r*)	Model 1 β	Model 2 β	Zero-order correlation (*r*)	Model 1 β	Model 2 β
DV: Current Self-Esteem						
Relatedness	.30***	.15***		.31***	.07	
Vividness	.29***	.08*		.41***	.17**	
Positivity	.45***	.36***		.56***	.45***	
DV: Future Self-Esteem						
Relatedness	.25***	.04	-.03	.31***	.04	.01
Vividness	.32***	.05	.02	.39***	.07	-.00
Positivity	.64***	.60***	.40***	.69***	.64***	.44***
Current Self-Esteem			.53***			.46***
DV: Hope						
Relatedness				.31***	.06	.03
Vividness				.45***	.25***	.19***
Positivity				.52***	.38***	.22***
Current Self-Esteem						.37***

*Notes*. Part III results derive from Sample 2; Part IV results derive from Sample 3. Variance inflation factors for all predictors in all models were less than 2.

* *p* < .05

** *p* < .01

*** *p* < .001.

**Table 6:** Multiple regression analyses of future self-identification components predicting visual imagery of future events and perceived temporal distance.

	Part III		Part IV	
	Zero-order correlation (*r*)	β	Zero-order correlation (*r*)	β
DV: VVIQ Graduation			
Relatedness	.12**	.02	.29***	.11
Vividness	.31***	.24***	.39***	.25***
Positivity	.26***	.14**	.35***	.20***
DV: VVIQ Post-Graduation			
Relatedness	.25***	.10*	.32***	.08
Vividness	.51***	.43***	.55***	.50***
Positivity	.33***	.10*	.28***	.02
DV: Perceived Temporal Distance				
Relatedness			-.22***	-.14*
Vividness			-.24***	-.17**
Positivity			-.14*	-.01

*Notes*. Part III results derive from Sample 1; Part IV results derive from Sample 3. Variance inflation factors for all predictors in all models were less than 2.

* *p* < .05

** *p* < .01

*** *p* < .001.

**Table 7:** Multiple regression analysis of future self-identification components predicting self-control.

	Part III		Part IV	
	Zero-order correlation (*r*)	β	Zero-order correlation (*r*)	β
Relatedness	.17***	.11*	.22***	.12*
Vividness	.21***	.12*	.25***	.17**
Positivity	.20***	.12*	.19***	.07

*Notes*. Part III results derive from Sample 1; Part IV results derive from Sample 3. Variance inflation factors for all predictors in all models were less than 2.

* *p* < .05

** *p* < .01

*** *p* < .001.

**Table 8:** Multiple regression analysis of future self-identification components predicting Grade Point Average (GPA) in Part III and GPA expectations in Part IV.

	Part III		Part IV	
	Zero-order correlation (*r*)	β	Zero-order correlation (*r*)	β
Relatedness	.10*	.12*	.14**	.17**
Vividness	-.01	-.03	-.01	-.09
Positivity	-.02	-.04	.04	.02

*Notes*. Part III results derive from Sample 1; Part IV results derive from Sample 3. Variance inflation factors for all predictors in all models were less than 2.

* *p* < .05

** *p* < .01

*** *p* < .001.

We next carried out multiple regression analyses with the three future self-identification components predicting current and future self-esteem separately. As predicted, the future self-identification components accounted for more variance in future self-esteem, *F*(3, 759) = 177.52, *p* < .001, *R*^*2*^ = .41, than current self-esteem, *F*(3, 752) = 71.73, *p* < .001, *R*^*2*^ = .23. Specifically, positivity was the strongest independent predictor of self-esteem, and its relationship was stronger with future self-esteem (β = .60) than with current self-esteem (β = .36). This pattern of results supports the concurrent relationship between future self-identification and self-esteem, with a stronger relationship between the positivity component and future self-esteem. Relatedness and vividness did remain significant predictors of current self-esteem after adjusting for the effect of positivity. However, these two predictors failed to reach significance for future self-esteem (see Table 5). Taken together, these results suggest that the three components of future self-identification can independently predict current levels of self-esteem, but that future self-esteem is more exclusively related to the positivity component.

One possible explanation for the above pattern of results is that the future self-positivity items simply measure a global self-evaluative construct. If this is the case, the strong relationship between the positivity component and self-esteem could just be due to a general tendency towards positivity. To test this possibility, a second model was performed with current self-esteem added as a predictor of future self-esteem (Table 5). The overall regression model was significant, *F*(4, 751) = 300.38, *p* < .001, *R*^*2*^ = .62. Importantly, positivity remained a significant unique predictor of future self-esteem (β = .40) adjusting for current self-esteem. These results suggest that the positivity component does not simply reflect a general positive self-disposition, but is strongly tied to future self-attitudes.

#### Visual imagery of future events

The correlations with the visual imagery variables were positive and significant for all three components (ranging from .12 to .51), with the strongest correlations being observed for vividness (see Table 6). The correlation coefficient for vividness was significantly higher than relatedness and positivity for VVIQ Post-Graduation (*z*s > 4.50, *p*s < .001). For VVIQ Graduation, the correlation coefficient for vividness and positivity were significantly higher than for relatedness (*z*s > 2.50, *p*s < .01), but they did not differ significantly from each other (*z* = 1.24, *p* = .22).

The future self-identification components accounted for significant variance in both VVIQ Graduation, *F*(3, 545) = 23.16, *p* < .001, *R*^*2*^ = .11, and VVIQ Post-Graduation, *F*(3, 545) = 69.00, *p* < .001, *R*^*2*^ = .28. The vividness component was the strongest unique predictor of visual imagery, with a stronger relationship found for VVIQ Post-Graduation (β = .43) than VVIQ Graduation (β = .24). This difference could be due to the VVIQ Post-Graduation items synching up with a similar timeframe as the future self-identification items (i.e., approximately 10 years in the future). Positivity still had significant unique relationships with both VVIQ Graduation (β = .14) and VVIQ Post-Graduation (β = .10), whereas relatedness only had a significant unique relationship with VVIQ Post-Graduation (β = .10). Taken together, these results support the concurrent relationship between future self-identification and visual imagery of future events, with a strong relationship between the vividness component and visual imagery. Furthermore, these results support the external convergent validity for the vividness factor because the future self-vividness and VVIQ items both involve imagination of the future.

#### Self-control

All three correlations between the components and self-control were positive and significant, and comparable in magnitude (ranging from .17 to .21; see Table 7). None of the correlation coefficients significantly differed from one another (*z*s < 0.75, *p*s > .50).

We next regressed self-control scores on the three future self-identification components. In concert, the three predictors accounted for significant variance in self-control, *F*(3, 541) = 12.69, *p* < .001, *R*^*2*^ = .07. Moreover, each component was a significant unique predictor of self-control, with comparable regression coefficients being observed for the components (see Table 7). This pattern of results demonstrates that all three components of future self-identification independently relate to self-control, suggesting an additive relationship amongst the three components of future self-identification in the domain of self-control.

#### Academic performance (college GPA)

Relatedness was the only component of future self-identification that correlated significantly with academic performance (*r* = .10, *p* < .05; see Table 8). Additionally, the correlation coefficient for relatedness was significantly different from the vividness and positivity correlation coefficients (*z*s > 2.00, *p*s < .05). When we regressed GPA on the three future self-identification components, the overall regression model was marginally significant, *F*(3, 520) = 2.08, *p* = .10, *R*^*2*^ = .01. However, the relatedness component remained a significant predictor of GPA (β = .12; see Table 8). These results suggest that perceiving relatedness between one’s current and future self is a positive, albeit modest, predictor of subsequent academic performance. Vividness and positivity of the future self were not significant predictors of GPA. The unique effect of relatedness remained statistically significant after adjusting for demographic factors (sex and race/ethnicity) and prior high school academic performance.

Robust academic achievement gaps are often reported in the literature [125–127]. Due to these achievement gaps, we conducted additional analyses to examine whether the observed relationships with GPA hold after controlling for certain demographic and background factors. We focused on two demographic variables (sex and race/ethnicity). Moreover, we investigated if the relationship between the relatedness component and subsequent college GPA held after controlling for previous academic performance using our records of students’ Calculated Index (sometimes referred to as the Colorado Index; CI) in Sample 1. CI is a composite of both high school GPA and standardized test scores (SAT or ACT). This composite is commonly used in college admissions.

The relatedness component remained a significant predictor of cumulative GPA when controlling for the demographic factors, *β* = .13, *t* = 2.69, *p* = .007. Furthermore, race was a significant predictor of GPA, *β* = .12, *t* = 2.72, *p* = .007, with White students earning higher GPAs than their non-White counterparts (which is consistent with the above-mentioned achievement gaps). In the regression model that added high school academic performance as a predictor, the relatedness component remained a significant predictor of cumulative GPA, *β* = .09, *t* = 2.07, *p* = .039. Race/ethnicity was no long a significant unique predictor of GPA, but there was a strong positive unique relationship between high school academic performance and cumulative college GPA, *β* = .46, *t* = 11.45, *p* < .001.

#### Path analytic results

**Fig 2:**
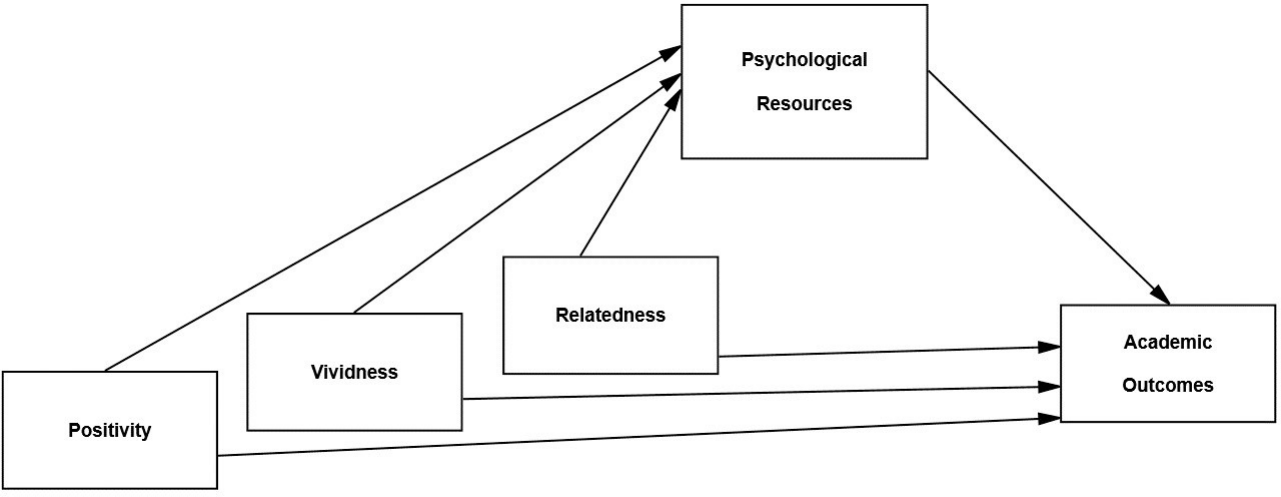
Path analytic models in Parts III and IV. Self-control, hope, or perceived temporal distance acted as intermediary variables (psychological resources) between the three future self-identification components and academic outcomes (cumulative GPA or semester GPA expectations). Covariances between the future self-identification components were included in all models.

To examine whether future self-identification relates to GPA indirectly through self-control, a path model was tested. The three future self-identification components were exogenous variables in the model; self-control and GPA were endogenous variables. Direct paths from the future self-identification components to GPA were included in the model, as well as indirect paths through self-control (see Fig 2 for a visualization of the path model). In this and all following path models, 95% bias-corrected confidence intervals stemmed from 5,000 bootstrapped samples [128]. The significance of the indirect effects was determined by ascertaining whether the 95% confidence intervals included zero or not.

The direct and indirect effects of the path model are included in Table 9. Both relatedness and self-control had significant direct effects on GPA. Nevertheless, all three future self-identification components had significant indirect effects on GPA through self-control. That is, for all three indirect effects, the 95% bias-corrected confidence intervals did not include zero (see Table 9). These results suggest that all three future self-identification components have indirect relationships with college GPA, with self-control identified as a possible intermediary variable.

**Table 9:** Path analytic results relating the future self-identification components to academic outcomes (cumulative GPA in Part III and semester GPA expectations in Part IV) through intermediary psychological resources (self-control, hope, or perceived temporal distance).

	Part III		Part IV	
	Coefficient	CI	Coefficient	CI
**Self-Control**				
*Direct Effects on DV*				
Relatedness	.08*	[.007, .148]	.07**	[.014, .118]
Vividness	-.03	[-.097, .040]	-.05	[-.097, .001]
Positivity	-.04	[-.119, .036]	.00	[-.056, .058]
Self-Control	.15**	[.055, .233]	.09**	[.025, .152]
*Indirect Effects*				
Relatedness	.01	[.002, .030]	.01	[.000, .021]
Vividness	.01	[.003, .030]	.01	[.002, .025]
Positivity	.01	[.002, .033]	.00	[-.001, .015]
**Hope**				
*Direct Effects on DV*				
Relatedness			.07**	[.014, .123]
Vividness			-.06*	[-.109, -.011]
Positivity			-.03	[-.096, .031]
Hope			.08***	[.032, .122]
*Indirect Effects*				
Relatedness			.01	[-.004, .021]
Vividness			.02	[.010, .046]
Positivity			.04	[.016, .068]
**Perceived Temporal Distance**				
*Direct Effects on DV*				
Relatedness			.07**	[.015, .119]
Vividness			-.04	[-.095, .005]
Positivity			.01	[-.050, .064]
PTD			-.00	[-.003, .000]
*Indirect Effects*				
Relatedness			.01	[-.000, .019]
Vividness			.01	[.000, .019]
Positivity			.00	[-.005, .007]

*Notes*. Part III results derive from Sample 1; Part IV results derive from Sample 3. Direct effects include the direct pathways between the future self-identification components and the intermediary variable on the dependent variable (cumulative GPA or GPA expectations). Indirect effects are the indirect pathways from the future self-identification components to the dependent variable through the intermediary variable. Coefficients are unstandardized path coefficients. Confidence Intervals (CI) are 95% bias-corrected confidence intervals based on 5,000 bootstrapped samples. Significance of indirect effects are based on the confidence intervals not including zero. Significance of direct effects are based on the presence of asterisk(s).

* *p* < .05

** *p* < .01

*** *p* < .001.

## Part IV: Future self-identification and psychological resources and outcomes (replication and extension)

Part IV had three study goals. The first goal was to replicate the above findings with a revised set of future self-identification items. The items were revised to rule out the possibility that method variance between items could have contributed to the identification of discriminant factors in the confirmatory factor analyses presented above. For instance, the previous future self-relatedness items used overlapping Euler circles to visually represent varying degrees of similarity and connectedness. This method variance may have contributed to the relatedness items being identified as a discriminant factor within the model. In Part IV, we removed the method variance between the future self-identification items to ensure that the three-factor structure was not driven by any prior variance between the items. To determine if the concurrent and predictive relationships observed above replicate with the revised future self-identification items, we included the dependent variables from Part III (self-esteem, visual imagery of future events, self-control, and academic performance).

In Part IV, because we did not have Institutional Analysis access to participants cumulative GPAs, academic performance was measured by a proxy variable, semester GPA expectations. Whereas some researchers have focused on mean differences between expected grades and actual grades [129], others have focused on the association between grade expectations and academic performance [130–132]. In the latter studies, a significant and positive relationship between expectations and academic performance has been consistently observed. These results demonstrate the utility of including an academic expectations variable (semester GPA expectations) as a proxy outcome variable, when direct access to earned GPA is not possible.

The second goal of Part IV was to extend the findings reported in Part III demonstrating relationships between the future self-identification components and psychological resources and academic outcomes. Prior research mainly focused on the relationship between relatedness of the future self and other variables of interest [8, 116]. In Part III of the current study, we expanded upon this research to support the inclusion of the positivity and vividness factors and demonstrated their external convergent validity with conceptually similar outcomes (i.e. self-esteem and vividness of visual imagery). However, the semantic overlap between the future self-positivity items and self-esteem and future self-vividness items and vividness of visual imagery, may have led us to overestimate the magnitude of these relationships. In Part IV, we included additional measures of psychological well-being (hope) and imagination of the future (perceived temporal distance of a future event) to better test their relationships with the future self-identification components in these two domains.

The State Hope Scale [133] measures goal-directed thinking. Hope is defined as “a cognitive set comprising agency (belief in one's capacity to initiate and sustain actions) and pathways (belief in one's capacity to generate routes) to reach goals” [133]. State hope is related to a variety of other well-being outcomes, including satisfaction with life [134] and depression [135]. The inclusion of state hope as a dependent variable in Part IV provides a stronger test of the relationship between future self-identification and psychological well-being, because there is less overlap between the future self-positivity items and the State Hope Scale as compared to the self-esteem scales. Goal-directed thinking is also relevant in academic environments. Specifically, hope is associated with higher levels of academic performance [136, 137]. Taken together, hope may provide an additional pathway through which future self-identification relates to academic outcomes.

The newly added imagination of the future variable was a measure of perceived temporal distance. The specific measure gauged participants’ rating of how close or far college graduation is perceived to be. Greater objective differences in time between current and future selves are associated with lower ratings of connectedness [6]. However, individuals differ in the extent distances in time are subjectively perceived [138]. Individuals who perceive time durations to be subjectively longer tend to discount delayed rewards at steeper rates [139]. In the present context, first-year college students who perceive college graduation to be a more distal event may be less likely to adopt behaviors in the present that lead to long-term academic success. The extent that the vividness component predicts the perceived temporal distance variable would help demonstrate the utility of including the component in the measurement of future self-identification.

The third goal of Part IV was to test whether the future-self vividness items simply measure a general tendency towards visual imagery use. One potential explanation for the relationship between the vividness component and the imagination of future events outcomes (e.g., the VVIQ variables), is that the future self-vividness items simply measure visual imagery use in general. If this is the case, the vividness component would not be a specific measure of future self-vividness (as originally designed). To rule out this explanation, we included a measure of general visual imagery use in Part IV. The Spontaneous Use of Imagery Scale (SUIS) [140] measures a general use of mental imagery in daily life, but does not assess vividness relating to future selves or events specifically. If the strength of the relationship between the vividness component and the SUIS is noticeably divergent from the relationships between vividness and the imagination of future events variables, it would be further external convergent and divergent validity of the vividness factor.

The inclusion of the above variables allowed us to further test the processes through which future self-identification influences academic outcomes. In Part III, we tested the indirect effects of the components of future self-identification on GPA through self-control. We extend these results in Part IV by testing whether indirect effects are found through the psychological well-being variable (state hope) and the imagination of the future variable (perceived temporal distance). These results served to further our understanding of the mechanisms that possibly underlie the relationship between future self-identification and academic outcomes.

### Method

#### Sample

Participants were a new sample of first-year college students who were 18 years or older (*N* = 348). The sample was 58% (*n* = 202) female. The racial and ethnic breakdown of the sample was as follows: 54.3% Caucasian (*n* = 189), 20.1% Hispanic or Latino (*n* = 70), 13.5% Asian (*n* = 47), 4.3% African American (*n* = 15), 1.7% Middle Eastern (*n* = 6), 0.3% Native American (*n* = 1), and 5.7% Other (*n* = 20). Data were collected from participants during the 8^th^ to 10^th^ week of their second semester in college.

#### Measures

*Future self-identification*. We made the following modifications to the future self-identification items used above to better control for method variance between items. First, we removed the overlapping Euler circles that accompanied the two relatedness component items. Second, we removed any wording in the items that references the overlapping circles. Third, the positivity item was transformed from a 1 to 100 scale to a 1 to 7 scale. See the S1 Appendix for a complete list of the modified items. Similar to Part III, all six future self-identification items were standardized and averaged together to create aggregate scores for the relatedness, vividness, and positivity components.

*Current and future self-esteem*. We used the same self-esteem measures as in Part III (current self-esteem Cronbach’s α = .883; future self-esteem Cronbach’s α = .865).

*Hope*. The State Hope Scale [133] was used as a measure of goal-directed thinking. An example item was “I can think of many ways to reach my current goals.” All six items were answered on an eight-point scale (1 = *Definitely False*, 8 = *Definitely True*). The internal consistency of the scale was good (Cronbach’s α = .859).

*Visual imagery of future events*. We used the same VVIQ measures as in Part III (VVIQ Graduation Cronbach’s α = .930; VVIQ Post-Graduation Cronbach’s α = .883).

*Perceived temporal distance*. Participants were presented with the following prompt: “Events may feel very close or may feel very far away, regardless of when they actually occur. Below, we would like you to rate how close or distant the following event feels to you. A value of 1 indicates that this event feels very close to you, a value of 100 indicates that this event feels very far away to you, and the other numbers represent intermediary levels of closeness/distance.” Participants then rated perceived distance to college graduation on the 1 to 100 slider scale. The mean response was 44.64 (*SD* = 25.27).

*Spontaneous use of imagery*. The Spontaneous Use of Imagery Scale (SUIS) [140] measures the degree that individuals use visual mental imagery in daily life. An example item was “When going to a new place, I prefer directions that include detailed descriptions of landmarks (such as the size, shape and color of a gas station) in addition to their names.” All 12 items were answered on a five-point scale (1 = *Never appropriate*, 5 = *Completely appropriate*). The internal consistency of the scale was acceptable (Cronbach’s α = .743)

*Self-control*. We used the same modified version of the Brief Self-Control Scale [124] as in Part III (Cronbach’s α = .741).

*GPA expectations*. To measure participants’ expectation of their current semester’s GPA, we provided them with the following prompt: “Combining all of your courses, what semester GPA do you expect to achieve?”. Participants provided their responses on a 0 to 4.33 slider scale. The maximum score was 4.33 (i.e., A+) because semester GPAs at the university can exceed 4.0. The mean response was 3.52 (*SD* = .38).

#### Procedure

The survey was administered online using Qualtrics software. Survey completion took less than 30 minutes and participants received 0.5 course credits for taking the survey.

### Results

The results section for Part IV is organized as follows. We first report the confirmatory factor analysis of the modified future self-identification items. We then report the unique relationships of the three future self-identification components and the variance they explain together in each domain of interest: psychological well-being (self-esteem, hope), imagination of the future (visual imagery of future events, perceived temporal distance), self-control, and semester GPA expectations. We conclude the Results section with path analytic models that probe the indirect effects of the future self-identification components on semester GPA expectations through the variables of self-control, hope, and perceived temporal distance.

#### Confirmatory factor analysis

The same three-factor model used in Parts I and II was fit to the Sample 3 data. See Table 3 for the standardized factor loadings. The fit of the model was acceptable, χ^2^(6) = 28.30, *p* < .001; CFI = .972; RMSEA = .103 [.067, .143], SRMR = .032. Moreover, the three-factor model significantly improved model fit over the one-factor and all two-factor models (ΔCFIs > .09). The three-factor model demonstrated good internal convergent validity, with the average variance extracted for the relatedness (.56), vividness (.78), and positivity (.59) factors all being above 0.5. Finally, good internal discriminant validity was also observed, with the squared correlations between the factors all being below the average variances extracted: relatedness and vividness (.33), relatedness and positivity (.28), and vividness and positivity (.30). These results demonstrate that removing the method variance between the future self-identification items did not prevent the identification of three discriminant factors within the future self-identification construct.

#### Psychological well-being

*Self-esteem*. Replicating the results in Part III, the three components together were significant predictors of or both current self-esteem, *F*(3, 344) = 59.24, *p* < .001, *R*^*2*^ = .34, and future self-esteem, *F*(3, 344) = 107.39, *p* < .001, *R*^*2*^ = .48 (see Table 5). Positivity had the strongest unique relationship with self-esteem, with a particularly strong effect observed for future self-esteem (β = .64). The only difference in patterns of results between Part III and Part IV is that the unique effect of the relatedness component on current self-esteem was not significant.

In Model 2, current self-esteem was added as a predictor of future self-esteem (Table 5). Positivity remained a significant unique predictor of future self-esteem (β = .44). Similar to the results in Part III, the Model 2 results demonstrate that the positivity component does not simply reflect a global self-evaluative construct; instead it appears to be particularly driven by degrees of positivity felt towards a future self.

*State hope*. All three future self-identification components significantly correlated with hope (ranging from .31 to .52; see Table 5). The correlation between hope and relatedness was significantly lower than for hope and both vividness and positivity (*z*s > 2.77, *p*s < .001). The coefficients for vividness and positivity with hope did not differ significantly (*z* = 1.45, *p* = .15). The overall regression model was significant, *F*(3, 344) = 56.28, *p* < .001, *R*^*2*^ = .33, with both vividness and positivity remaining significant unique predictors of hope. Holding a more vivid imagination of a future self, as well as having a higher degree of positivity toward the future self, is associated with more goal-directed thinking. These significant relationships held in a second regression model that added current self-esteem as a predictor.

#### Imagination of the future

*Visual imagery of future events*. The prediction of the VVIQ outcomes by the future self-identification components can be seen in Table 6. The future self-identification components accounted for significant variance in both VVIQ Graduation, *F*(3, 344) = 28.17, *p* < .001, *R*^*2*^ = .20, and VVIQ Post-Graduation, *F*(3, 344) = 50.77, *p* < .001, *R*^*2*^ = .31. Vividness had the strongest unique relationship with both VVIQ Graduation (β = 43) and VVIQ Post-Graduation (β = .50). These patterns largely replicated the findings observed in Part III, with the only difference being that the small unique effects of relatedness and positivity on VVIQ Post-Graduation were no longer significant.

*Perceived temporal distance*. All three future self-identification components correlated negatively with perceived temporal distance (ranging from -.14 to -.24; see Table 6). None of the correlation coefficients significantly differed from one another (*z*s < 1.87, *p*s > .05). The overall regression model was significant, *F*(3, 344) = 8.80, *p* < .001, *R*^*2*^ = .07, with relatedness and vividness remaining significant unique predictors of perceived temporal distance. These negative relationships imply that perceiving relatedness between the present and future self, as well as being able to vividly imagine the future self, is associated with perceiving college graduation to be closer in time.

*Spontaneous use of imagery*. To ensure that the future self-vividness items do not simply measure a general tendency towards the use of visual imagery, we tested the extent the future self-identification components predicted the use of visual imagery in daily life (the SUIS). The overall regression model was significant, *F*(3, 344) = 4.62, *p* < .01, *R*^*2*^ = .04. However, none of the three components were significant unique predictors of the SUIS (βs < .12, *p*s > .05). Moreover, when the SUIS was added to multiple regression analyses predicting the three imagination of the future variables, vividness remained a significant unique predictor of VVIQ Graduation (β = .22, *p* < .001), VVIQ Post-Graduation (β = .48, *p* < .001), and perceived temporal distance of college graduation (β = -.17, *p* < .01). These results help demonstrate that the future self-vividness items are not simply a measure of visual imagery tendencies. Instead, they appear to be particularly attuned to vividness relating to future selves.

#### Self-control

The magnitude of the correlations between the three components and self-control were similar (see Table 7; *z*s < 1.13, *p*s > .05). Together, the three components predicted self-control, *F*(3, 344) = 10.15, *p* < .001, *R*^*2*^ = .08. However, in contrast to Part III, only relatedness and vividness were unique predictors of self-control.

#### Semester GPA expectations

The pattern of relationships between the future self-identification components and semester GPA expectations replicated the patterns in Part III that focused on cumulative GPA as the dependent variable (see Table 8). The overall regression model was significant, *F*(3, 344) = 2.94, *p* < .05, *R*^*2*^ = .03. However, relatedness was the only significant predictor of semester GPA expectations (β = .17). Similar to the cumulative GPA results in Part III, semester GPA expectations appear to be directly related to future self-identification due to the perceived relatedness between current and future selves. This unique effect of relatedness remained statistically significant after adjusting for demographic factors (sex and race/ethnicity).

We performed an additional regression analysis with demographic factors included as predictors (sex and race/ethnicity) of GPA Expectations. We did not have access to high school academic performance in Sample 3. The relatedness component remained a significant predictor of GPA Expectations when controlling for the demographic factors, *β* = .17, *t* = 2.85, *p* = .005. Furthermore, sex was a significant predictor, *β* = -.12, *t* = 2.31, *p* = .022, with males reporting lower GPA Expectations compared to females. Race/ethnicity predicted GPA expectations only marginally in this model (*p* = .091), showing a trend that White students expected higher GPA than their non-White counterparts.

#### Path analytic results

The following path analytic models tested the degree to which the three future self-identification components had indirect effects on academic performance. The visualization of the path models can be seen in Fig 2. In the first model, self-control acted as the intermediary variable between the three future self-identification components and GPA expectations. In the second and third models, hope and perceived temporal distance acted as the intermediary variable, respectively. The former model served as a replication of the path model in Part III that included cumulative GPA as the dependent variable. The latter two models tested the extent future self-identification relates to academic performance through psychological well-being and perceived temporal distance variables.

Table 9 includes the direct and indirect effect estimates of the three path models. Focusing first on the model that included self-control as the intermediary variable, both relatedness (*b* = .07, *p* < .01) and self-control (*b* = .09, *p* < .01) had significant direct effects on GPA expectations. Moreover, the 95% confidence intervals for both the relatedness and vividness indirect effects did not include zero. Though future self-vividness did not directly influence GPA expectations, it had an indirect effect through increased levels of self-control. The relatedness component influenced GPA expectations both directly and through increased levels of self-control.

The second path model included hope as the intermediary variable between the future self-identification components and GPA expectations. In this model, the 95% confidence intervals for both vividness and positivity indirect effects did not include zero. Higher vividness and positivity towards the future self was associated with increased state hope, which was associated with higher semester GPA expectations.

The third model included perceived temporal distance as the intermediary variable. The 95% confidence interval for only the vividness indirect effect did not include zero. Higher vividness influenced semester GPA expectations through perceiving graduation as closer in time. Taken together, the above path analytic results suggest that the three components of future self-identification can relate to academic outcomes through a variety of mechanisms, including self-control, psychological well-being (state hope), and imagination of future events (perceived temporal distance of college graduation). However, future longitudinal research will be needed to replicate these indirect effect analyses to better identify the temporal order of these cross-sectional effects.

## General discussion

### Summary of findings

The present findings show that the three components of future self-identification—relatedness, vividness, and positivity toward one’s future self—represent correlated but independent factors. Most prior measures of future self-identification focused on only one or two of these components [but see 21, 92]. As a result, it remained unclear which aspects of future self-identification were associated with psychological and behavioral outcomes. The results of the current research suggest that the answer to this question is domain dependent, which demonstrates the utility of measuring all three components in future studies.

For self-control, all three components of future self-identification had significant relationships, with the strength of the correlation and regression coefficients being comparable among the three components (though the unique effect of positivity was not significant in Part IV). These results suggest an additive model of the effects of the components of future self-identification for the domain of self-control, in which higher levels of each future self-identification component are uniquely associated with increased self-control. As the path analytic models further demonstrated, the relationship between the future self-identification components and self-control has particular relevance for the academic domain, as significant indirect effects were observed between the components and grade expectations and performance.

In other domains, one of the three components had a noticeably stronger relationship than the other two. These distinctive relationships demonstrate the external convergent validity of the components and the utility of adopting a three-component model of future self-identification. For self-esteem, the positivity component had the strongest relationships compared to relatedness and vividness, with a particularly strong relationship observed between positivity and self-esteem towards the future self. These results support and extend prior research that demonstrated an association between future self-identification and psychological and subjective well-being [7, 116]. Yet, by not taking the positivity component into account, prior research may have overestimated the degree of relationship between future self-relatedness and well-being. Finally, the positivity component does not simply measure a global positive self-evaluation. In models predicting future self-esteem and state hope, the positivity component remained a significant unique predictor even after adjusting for current self-esteem, suggesting that the positivity component taps into the valence attached to future selves.

As expected, visual imagery of future events was most strongly related to the vividness component, though positivity noticeably related to vivid imagery of certain future events as well (e.g., college graduation). Individuals with higher positivity towards future selves may be more likely to spend time imagining the vivid details surrounding happy and celebratory events in the future. The extent that positivity towards future selves relates to vivid imagery of future events that span a range of perceived affective states remains an open question. In Part IV, we included an additional imagination of the future variable, which measured perceived temporal distance of college graduation. For this outcome, both vividness and relatedness negatively predicted perceived temporal distance. Holding a vivid conception of a future self and perceiving relatedness between current and future selves are associated with perceiving college graduation as closer in time. Perceiving future events as being closer in time might be particularly relevant for academic outcomes, due to subjective time perception relating to intertemporal preferences [138]. Individuals who perceive time durations to be subjectively longer tend to discount delayed rewards at steeper rates [139]. The current results suggest that perceiving graduation as closer in time might confer academic benefits, as the vividness component was shown to relate to semester GPA expectations through perceived temporal distance.

Finally, academic outcomes (cumulative GPA and semester GPA expectations) were only directly related to the relatedness component. This latter finding extends previous research by showing that relatedness accounts for variance in academic performance net of the vividness and positivity components. Vividness and positivity did demonstrate indirect effects on academic outcomes, however. These results suggest that all three future self-identification components may be relevant to academic performance, but vividness and positivity possibly exert their influences through other psychological variables. The present study identified three potential intermediary psychological variables (self-control, hope, and perceived temporal distance). But these preliminary results warrant replicability, as well as identification of other intervening variables between future self-identification and academic performance.

With self-control having a significant relationship with academic outcomes, it may be surprising that the vividness and positivity components had significant relationships with self-control but not significant direct relationships with GPA. While all three components tap into individuals’ perceptions of their future self, the relatedness component is the only one explicitly assessing the link between current and future selves; the vividness and positivity items only measure individuals’ perceptions of their future self with no mention of the current self. Being a student is likely to be a salient identity of emerging adults who are experiencing their first year at college. Consequently, academic performance may therefore be more directly tied to the relatedness component because this component taps into their current self-identity as a student.

According to the typology of mediation effects proposed by Zhao, Lynch, and Chen [141], the significant indirect effects of the vividness and positivity components on academic performance would be termed *indirect-only mediation* effects. These effects are still meaningful in the absence of significant direct or total effects. These findings suggest that merely having strong future self-identification may not always lead to positive outcomes such as better academic performance. Some individuals with a vivid and positive future perception may direct their effort to goal-directed behaviors (i.e., self-control) and in turn perform better in school (i.e., higher GPA). However, others with a vivid and positive future perception may daydream about a positive future without taking action or exercising self-control and do not do well. These opposing indirect effects could then cancel each other out with regards to the total effects of vividness and positivity on academic performance. These findings highlight the importance for future research to address the conditions under which future self-identification enhances performance.

Future longitudinal research will be necessary to extend these indirect effect analyses to more conclusively tease apart the temporal order and causal nature of the relationships. The long-term associations between future self-identification and later behavioral/psychological outcomes is a relatively unexplored topic. In a recent article, Reiff, Hershfield, and Quoidbach [142] found that the relatedness component was positively correlated with a measure of well-being (life satisfaction) ten years later. The current results demonstrated that the future self-identification components had different unique relationships with psychological well-being (e.g., self-esteem, hope). As a result, it is important for future research to test longitudinal relationships between all three future self-components and well-being, as well as investigate the mechanisms that underpin these longitudinal associations.

### Implications, limitations, and future directions

To test the conceptualization of future self-identification as a triadic construct, our first aim was to develop a more comprehensive measure of future self-identification. We built upon and extended current measures of the construct by including items designed to tap into all three components. Through confirmatory factor analysis, we demonstrated that the three components could be measured as discriminant factors within an overall model. We further reported on the test-retest reliability over a period of five weeks and replicated the model in two additional samples. These results helped establish the reliability and internal validity of the future self-identification measure and its utility for researchers seeking to assess future self-identification in a more nuanced way.

A better understanding of the multi-dimensionality of future self-identification is relevant to intervention research. Recent interventions were designed to increase levels of future self-identification [8, 80, 90]. The goal of these interventions was to foster long-term beneficial behavior such as monetary savings [90] and delinquency [91, 92] by increasing feelings of connection between present and future selves. The current research suggests that the component most relevant to an intervention varies with the type of outcome of interest. Consequently, interventions should focus on the component(s) of future self-identification that have the strongest relationship in the outcome being targeted. For example, if the positivity component has the strongest relationship with a target outcome (e.g., psychological well-being), interventions should focus on positivity rather than relatedness or vividness. Ensuring that interventions are tailored to the most relevant component(s) will be necessary during the planning and design process of subsequent future self-identification interventions. It also might be the case that other components of future self-identification will be identified. Though the current review and analyses supported three components of future self-identification [4], future research will need to explore the possibility of other components.

Another direction for future research is to determine whether the components of future self-identification exert interaction effects on outcomes. We did not have any *a priori* hypotheses about interaction effects, but we believe extending our findings along these lines is an important next step. For instance, certain outcomes may require a high degree of future self-identification on multiple components. For example, the benefit conferred by a vivid future self on attainment of goals is likely to vary with the valence of the future self. Whereas a vividly imagined positive future may elicit behaviors instrumental to goal attainment, a vividly imagined negative future may evoke self-defeating behaviors due to negative psychological states (e.g., dread) that undermine goal attainment.

Some limitations of the current study should be noted. First, each of the three future self-identification factors was measured with only two indicators. There are statistical benefits to including more than two items per factor when carrying out a factor analysis, such as enhancing reliability [143]. For this reason, we carried out multiple methods of measuring reliability and validity (both internal and concurrent) of the factor model, such as testing the factor structure over multiple time points and across different samples of participants. The findings from this investigation demonstrate that the three components of future self-identification can be measured with a brief scale shown to be both reliable and valid. Nevertheless, developing future self-identification subscales with more items per factor is an important avenue for future research. Having brief and longer measures of each factor enables researchers to choose between the statistical benefits of assessing each factor with more items and the need to measure variables parsimoniously when respondent burden is an issue (e.g., multi-disciplinary longitudinal studies). Moreover, to further validate the future self-identification components, future research should include measures that utilize a variety of method formats. The current future self-identification and psychological resource measures relied exclusively on self-report, so our understanding of the consequences of future self-identification will be aided by a wider diversity of measurement methodologies.

A second limitation is that the current study focused solely on undergraduates in their first year of college. The choice of this sample was pertinent because adolescents entering college are involved in a major life transition. During this stage, feelings toward one’s future self may be particularly important in fostering long-term beneficial behaviors (e.g., academic persistence). Yet, the extent that the three-factor model of future self-identification observed in the current samples generalizes to other age or cultural groups is unknown. Even within the college student population, entering freshmen may not have as much information about their future self with a college degree as compared to more advanced students. It will be fruitful for future longitudinal research to test whether there are changes in the factor structure of future self-identification during college, and the psychological and academic consequences of these changes. The concurrent and predictive validity of the three components might also differ based on age group, the likelihood of life transitions in the near future, and cultural context. For instance, future self-identification may be especially relevant when individuals go through role transitions. The current study focused on students starting college, but other relevant important life transitions could include starting a family, getting divorced, receiving a diagnosis of a chronic disease, re-careering, and retirement. Future research that targets these different populations will help elucidate the critical role that future self-identification plays across different life stages and transitions.

It will be important for future research to investigate whether the effects of future self-identification are moderated by factors such as demographic characteristics. One such example is whether the relationship between future self-identification and academic performance differs across racial/ethnic groups, due to the academic achievement gaps often reported in the literature [125–127]. Cultural backgrounds and experiences may impact the way students conceptualize their future selves, as well as how they perform academically. For instance, college generation status (first-generation vs. continuing-generation students) was shown to moderate the relationship between temporal psychological factors and psychological resources [5]. Demographic groups that differ in college generation status, as well as on other cultural and historical factors, may identify with their post-college future self differently. Therefore, the pathways by which future self-identification impacts academic behavior and performance may vary across demographic groups. We believe this is a promising avenue for future investigation.

Consistent with previous research, we found that the components of future self-identification were related to several psychological resources (e.g., self-control, self-esteem) which influence a wide range of consequential outcomes [124]. Nevertheless, a third limitation of the present study is that we assessed only one behavioral outcome, with future self-identification accounting for a small proportion of variance in cumulative GPA. Prior research has demonstrated an effect of future self-identification on a variety of behavioral outcomes, including savings behavior [21], exercise rates [64], and delinquency [92]. Thus, future research is warranted that investigates the unique relationship between the three future self-identification components and behavioral outcomes such as work productivity and adherence to medical regimens.

## Conclusions

Our overarching aim was to increase the cohesiveness of research on the future self by (a) reviewing the literature and addressing conceptual differences about construct labels, (b) formally testing a measure of future self-identification components, and (c) examining the predictive validity of the three components of future self-identification. We relabeled the global construct (future self-identification as opposed to future self-continuity) and one component (relatedness in place of similarity). Our studies provided support for a three-component model of future self-identification [4] and provided preliminary evidence of the validity of these component measures: (1) perceived *relatedness* between current and future selves, (2) the degree of *vividness* when future selves are imagined, and (3) the degree of *positivity* felt toward the future selves. Because the influence of the three components varied across outcomes, researchers should consider the multi-dimensional structure of future self-identification in formulating hypotheses about its relationships with outcomes of interest, and in designing and evaluating interventions that target the enhancement of future self-identification.

## Supporting information

S1 File Read me document for the data files. (DOCX)

S2 File Sample 1 data file in.csv format. (CSV)

S3 File Sample 1 data file in.sav format. (SAV)

S4 File Sample 2 data file in.csv format. (CSV)

S5 File Sample 2 data file in.sav format. (SAV)

S6 File Sample 3 data file in.csv format. (CSV)

S7 File Sample 3 data file in.sav format. (SAV)

S1 Appendix Future self-identification scale. (DOCX)
