# Supplementary material for: A test of a triadic conceptualization of future self-identification
Source: PLoS One. 2020 Nov 24;15(11):e0242504. doi: 10.1371/journal.pone.0242504 (PMC7685460; doi:10.1371/journal.pone.0242504)
Supplement: S1 File — (DOCX) [file pone.0242504.s001.docx]

Read Me – Data Files

Csv and SPSS data files are included for each of the three samples included in the study (Sample1, Sample2, Sample3).

Below is a list of the variables included in the data set with brief descriptions.

**ID**: Subject identifier (called “SubjectID in the Sample3 files)

**Sex**: 0 = female, 1 = male

**Race**: 1 = White/Caucasian/European American, 2 = East Asian/Asian American/Southeast Asian/Pacific Islander, 3 = Hispanic/Latino(a)/Chicano(a)/Latin American, 4 = South Asian/Indian, 5 = Middle Eastern/Arab/Arab American, 6 = Native American/Alaska Native, 7 = Black/African American/African/West Indian, 8 = other

Future Self-Identification Items

**Similarity**

**Connectedness**

**Clarity**

**EaseOfVisualization**

**Liking**

**Valence**

In the Sample1 data files, there are future self-identification items from Time 1 and Time 2. The Time 1 data have T1 after the above six items’ names; the Time 2 data have T2 after the above six items’ names.

Intrapsychic Resources and Academic Outcomes

**VVIQ_Graduation** (Sample1Data T1, Sample3Data)

**VVIQ_PostGraduation** (Sample1Data T1, Sample3Data)

**SelfControl** (Sample1Data T1, Sample3Data)

**CurrentSelfEsteem** (Sample2Data, Sample3Data)

**FutureSelfEsteem** (Sample2Data, Sample3Data)

**Hope** (Sample3Data)

**PerceivedTemporalDistance** (Sample3Data)

**SUIS** (Sample3Data)

**CumulativeGPA** (Sample1Data)

**GPAExpectations** (Sample3Data)
