# Supplementary material for: A test of a triadic conceptualization of future self-identification
Source: PLoS One. 2020 Nov 24;15(11):e0242504. doi: 10.1371/journal.pone.0242504 (PMC7685460; doi:10.1371/journal.pone.0242504)
Supplement: S1 Appendix — (DOCX) [file pone.0242504.s008.docx]

**Appendix**

**Future Self-Identification Scale**

A full list of the prompts and response scales for the future self-identification measure. The prompts and response scales refer to the modified items used in Part IV. See the method section of Part I for a description of the original items.

| Components and Items | Prompt | Response Scale |
| --- | --- | --- |
| *Relatedness* |  |  |
| Similarity | Please select how similar you feel to your future self? | 1 = not at all similar to my future self  7 = very similar to my future self |
| Connectedness | Please select how connected do you feel to your future self? | 1 = not at all connected to my future self,  7 = very connected to my future self |
| *Vividness* |  |  |
| Clarity | When you imagine your future self, how vividly do you picture it? | 1 = not at all vividly; I do not have a clear image in my head of my future self  7 = very vividly; I have a very clear image in my head of my future self |
| Ease of  Visualization | How easy is it for you to visualize a mental picture of your future self? | 1 = Very difficult  7 = Very easy |
| *Positivity* |  |  |
| Liking | How much do you like your future self? | 1 = don't like at all  7 = like as much as possible |
| Valence | Please characterize your future | 1 = Very negative  7 = Very positive |
